# Efficacy and Safety of Intracoronary versus Intravenous Administration of Tirofiban during Percutaneous Coronary Intervention for Acute Coronary Syndrome: A Meta-Analysis of Randomized Controlled Trials

**DOI:** 10.1371/journal.pone.0129718

**Published:** 2015-06-11

**Authors:** Xiuying Tang, Runjun Li, Quanmin Jing, Yingfeng Liu, Peng Liu

**Affiliations:** 1 Department of Cardiology, The first hospital of QinHuangDao, QinHuangDao, HeBei, China; 2 Department of Emergency Medicine, The first hospital of QinHuangDao, QinHuangDao, HeBei, China; 3 Department of Cardiology, General Hospital of Shenyang Military Area Command, Shenyang, LiaoNing, China; 4 Department of Cardiology, Zhujiang hospital, Southern Medical University, GuangZhou, GuangDong, China; University of Louisville, UNITED STATES

## Abstract

**Background:**

Percutaneous coronary intervention (PCI) is known as the most effective treatment for acute coronary syndrome (ACS). However, without proper therapy and patient management, stent thrombosis after PCI may lead to another myocardial infarction. In addition to aspirin and clopidogrel, tirofiban is often used as an antiplatelet therapy in patients with ACS. To date, there has been no comprehensive evaluation of the efficacy and safety of intracoronary (IC) tirofiban administration for ACS patients undergoing PCI compared with intravenous (IV) administration. Therefore, this meta-analysis was conducted to investigate the clinical efficiency and safety of IC versus intravenous (IV) tirofiban in ACS patients undergoing PCI.

**Methods:**

We searched PubMed and Medline for randomized controlled trials (RCTs) comparing IC versus IV administration of tirofiban in ACS patients undergoing PCI. We evaluated the effects of tirofiban on thrombolysis in myocardial infarction (TIMI) grade 3 flow after PCI, TIMI myocardial perfusion grade 3 (TMP grade 3), left ventricular ejection fraction (LVEF), major adverse cardiovascular events (MACE), target vessel revascularization (TVR), death, reinfarction and adverse drug effects (specifically bleeding events).

**Results:**

Seven trials involving 1,027 patients were included in this meta-analysis. IC administration of tirofiban significantly increased TIMI grade 3 flow (OR 2.11; 95% CI 1.02 to 4.37; P = 0.04) and TMP grade 3 (OR 2.67; 95% CI 1.09 to 6.49; P = 0.03, I^2^ = 64%) while reducing MACE (OR 0.46, 95% CI: 0.28 to 0.75; P = 0.002) compared with IV administration of tirofiban. No significant differences were observed in the occurrence of TVR, death, reinfarction and the incidence of bleeding events between the two groups.

**Conclusions:**

This meta-analysis supports the use of IC over IV administration of tirofiban in patients with ACS to improve TIMI flow, TMP flow and MACE. However, there was no statistically significant difference in the risk of bleeding complications between the two groups.

## Introduction

Due to high morbidity, mortality, readmissions and high costs, acute coronary syndrome (ACS) is the most severe form of coronary artery disease. Currently, percutaneous coronary intervention (PCI) is the most effective treatment for ACS [1–4]. However, without proper therapy and patient management, stent thrombosis after PCI may lead to another myocardial infarction.

Dual antiplatelet therapy with aspirin and clopidogrel plays a crucial role in the management of ACS patients undergoing PCI [5–8]. This therapy is also associated with a reduction in recurrent ischemic events after ACS and prevention of stent thrombosis following PCI [9–12]. However, some studies have shown that certain patients with ACS are resistant to aspirin and/or clopidogrel [13–17]. In addition to the high risk of ACS, recurrent thrombotic events may continue to occur, despite the use of standard dual antiplatelet treatment regimens. Therefore, GPIIb-IIIa inhibitors (GPIs) are often used as antiplatelet therapy in addition to aspirin and clopidogrel in patients with ACS because of their distinctive and complementary mechanisms of inhibition [8,18].

Several studies and meta-analyses have demonstrated that abciximab provides measurable benefits, such as a reduction of adverse cardiovascular events (reinfarction), including death from any cause during follow-up [19–21]. In addition, other studies and meta-analyses have indicated that the administration of IC versus IV abciximab is potentially even more beneficial [22–25] in terms of decreasing 30-day mortality rates, target vessel revascularization (TVR) [22,24] and major adverse cardiovascular events (MACE) [24] as well as increasing TIMI grade 3 flow [24,25] and 6-month left ventricular ejection fraction (LVEF) [23]. However, the use of small molecules (tirofiban) is an attractive strategy because of their ability to reverse the inhibition of platelet aggregation and the associated lower costs.

As mentioned above, there is little doubt about the benefits of IC compared with IV administration of abciximab. Whether IC tirofiban offers the same benefits as abciximab remains to be determined. One meta-analysis [26] indicated that compared with the commonly applied care regime, IV tirofiban tended to reduce the risk of 30-day and/or 6-month MACE for patients with ACS, which are results that are generally in line with a recent Cochrane review [27]. Compared with IV administration, there has been no comprehensive evaluation of the efficacy and safety of IC tirofiban for ACS patients undergoing PCI. Thus, this meta-analysis of RCTs was conducted to evaluate the efficacy and safety of IC versus IV administration of tirofiban in ACS patients undergoing PCI.

## Study Design and Methods

We used a predesigned protocol for the literature search, study selection and data synthesis. We adhered closely to the Preferred Reporting Items for Systematic Reviews and Meta-Analyses (PRISMA) guidelines [28].

### Search Strategy

Two reviewers (T. XY, L. RJ) independently conducted a thorough search of the PubMed and Medline databases for reports of all RCTs conducted up to May 2014 on the clinical outcomes of IC versus IV administration of tirofiban in ACS patients undergoing PCI. The Medical Subject Headings search strings for this literature search were as follows: (1) “myocardial infarction” OR “acute coronary syndrome” AND “intracoronary” AND”intravenous” AND “GPIs” OR “GPIIb-IIIa inhibitors” OR “tirofiban” Filtering “clinical trial”, “Humans” and “English”; (2) “Intracoronary [All Fields] and intravenous [All Fields] and tirofiban [All Fields]”; (3) “Intracoronary [All Fields] and intravenous [All Fields] and GPIIb-IIIa inhibitors [All Fields]”; and (4) “Intracoronary [All Fields] and intravenous [All Fields] and GPIs [All Fields]”. The language was limited to English or Chinese. We also manually searched clinicaltrials.gov for potential RCTs that were not published, but we unfortunately did not find any trials that met the eligibility criteria. At same time, we reviewed the relevant meta-analyses and references to ensure that all pertinent studies were included in the preliminary review.

### Selection Criteria

The inclusion criteria were as follows: 1. ACS Patients undergoing PCI; 2. RCTs; 3. IC versus IV tirofiban; and 4. reporting of at least one of the criteria for determining the efficacy, safety or outcomes of tirofiban, which consisted of TIMI grade 3 flow, LVEF, MACE, TVR, death and bleeding events. Two reviewers (T. XY and L. RJ) independently screened all of the potential studies from the electronic search to assess their eligibility for inclusion. Disagreements were settled by consulting with a third reviewer (J. QM).

### Data Extraction and Synthesis

Data extraction was independently undertaken by two reviewers (L. YF, L. P) using a predesigned data collection form. They independently extracted the following details of the studies: publication details (such as the first author’s last name, year of publication and country in which the study was performed); trial information (such as the study design, inclusion criteria, number of patients, intervention and follow-up); patient characteristics (such as age, gender and medical therapy); and outcome measures (such as TIMI grade 3 flow, TMP grade 3, LVEF, MACE, TVR, death and bleeding events). Disagreements were resolved by consulting with the third reviewer (J. QM).

### Quality Assessment

Two reviewers (L. P, L. RJ) independently appraised study quality by estimating the risk of bias in the included trials in six domains based on the Cochrane Collaboration’s tool for assessing the risk of bias. The assessments of the judged outcomes were explicitly as follows:

- Low risk of bias;
- High risk of bias; and
- Unclear risk (where there was lack of information or uncertainty regarding the potential for bias).

Discrepancies were resolved by consultation with the third reviewer (J. QM).

**Fig 1:**
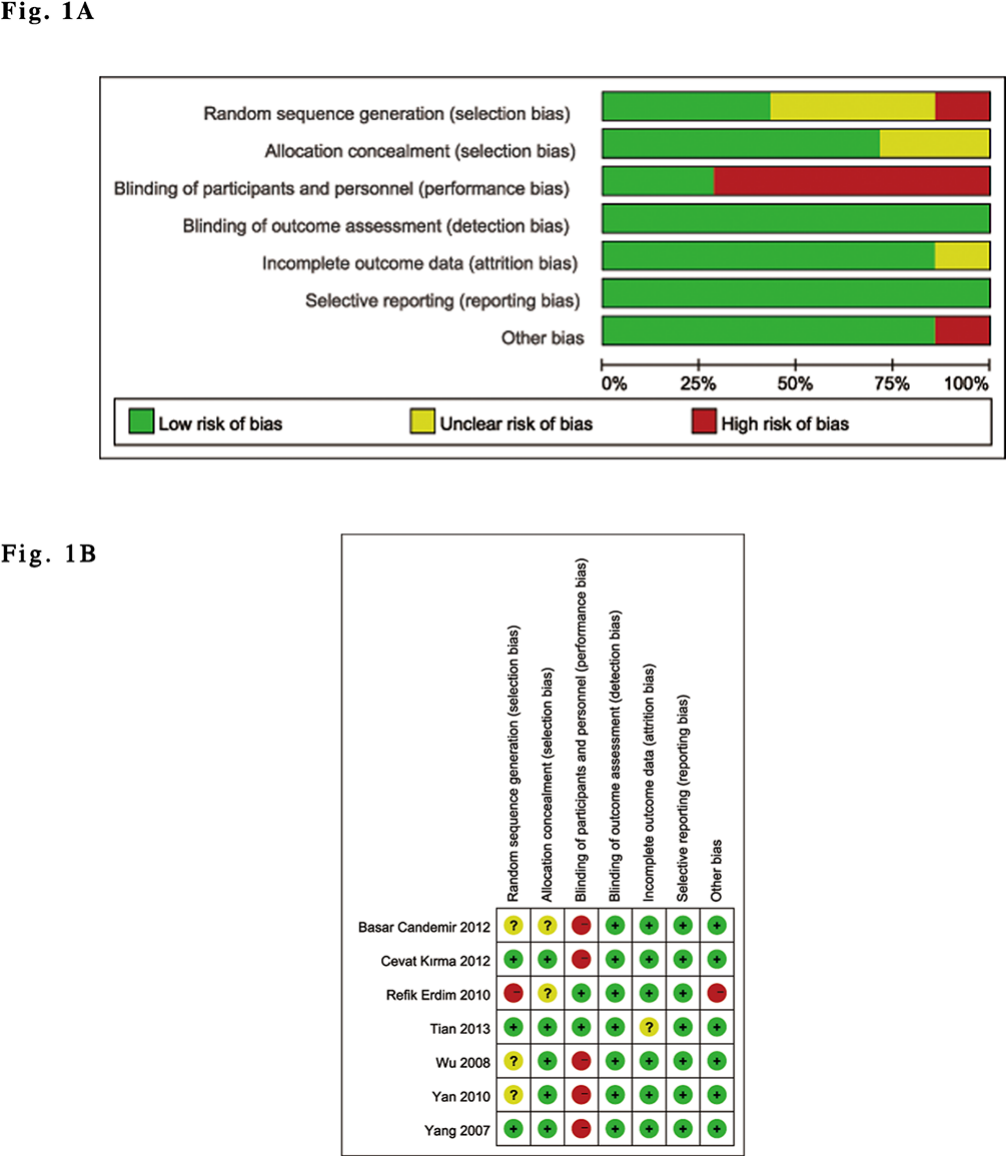
Risk of bias summary and graph. (A). Risk of bias summary: review of the authors' judgments about each risk of bias item for each included study. (B). Risk of bias graph: review of the authors' judgments about each risk of bias item, presented as percentages across all of the included studies.

Assessments of risk of bias are described in the risk of bias table for each included trial, as indicated in Fig 1 and Table 1.

**Table 1:** Design characteristics of included studies.

Studies	Random sequences	Allocation concealment	Blinding proceduces	Compliance description	Attrition description	Analysis approaches
Tian et al.2013	computer-generated random-allocation system	Yes	Double blinding	Yes	NA	Perprotocol
Candemir et.al,2012	NA	NA	Open trial	Yes	Yes	Perprotocol
Kırma et.al,2012	Sealed unlabeled envelopes	Yes	Open trial	Yes	Yes	Perprotocol
Erdim et.al,2010	NA	NA	Double blinding	Yes	Yes	ITT
Yan et al.2010	NA	Yes	Open trial	Yes	Yes	Perprotocol
Wu et.al,2008	NA	Yes	Open trial	Yes	Yes	Perprotocol
Yang et.al,2007	The random number table method	Yes	Open trial	Yes	Yes	Perprotocol

NA = not available; ITT = intention-to-treat.

### Statistical Methods

The statistical analyses were performed using Review Manager 5.2. For dichotomous data, the results are presented as the odds risk (OR) with 95% confidence intervals (CIs). Continuous outcomes are presented as mean differences (MDs) or standardized mean differences (SMDs) in both the IC and IV treatment groups. Heterogeneity among studies was determined using the Chi square-based Q test and the I^2^ statistic. The data were pooled using a fixed-effects model unless substantial heterogeneity was observed (I^2^≥50% and heterogeneity P≤0.1), in which case a random-effects model was employed. Potential publication bias for each of the pooled study groups was evaluated using a funnel plot. Again, simple pooling of the sensitivity analysis was conducted to test the sensitivity of the results of the systematic review and the meta-analysis methodology and to obtain more credible results regarding test performance. A two-tailed P value of less than 0.05 was considered statistically significant.

## Results

### Selected studies and characteristics

**Fig 2:**
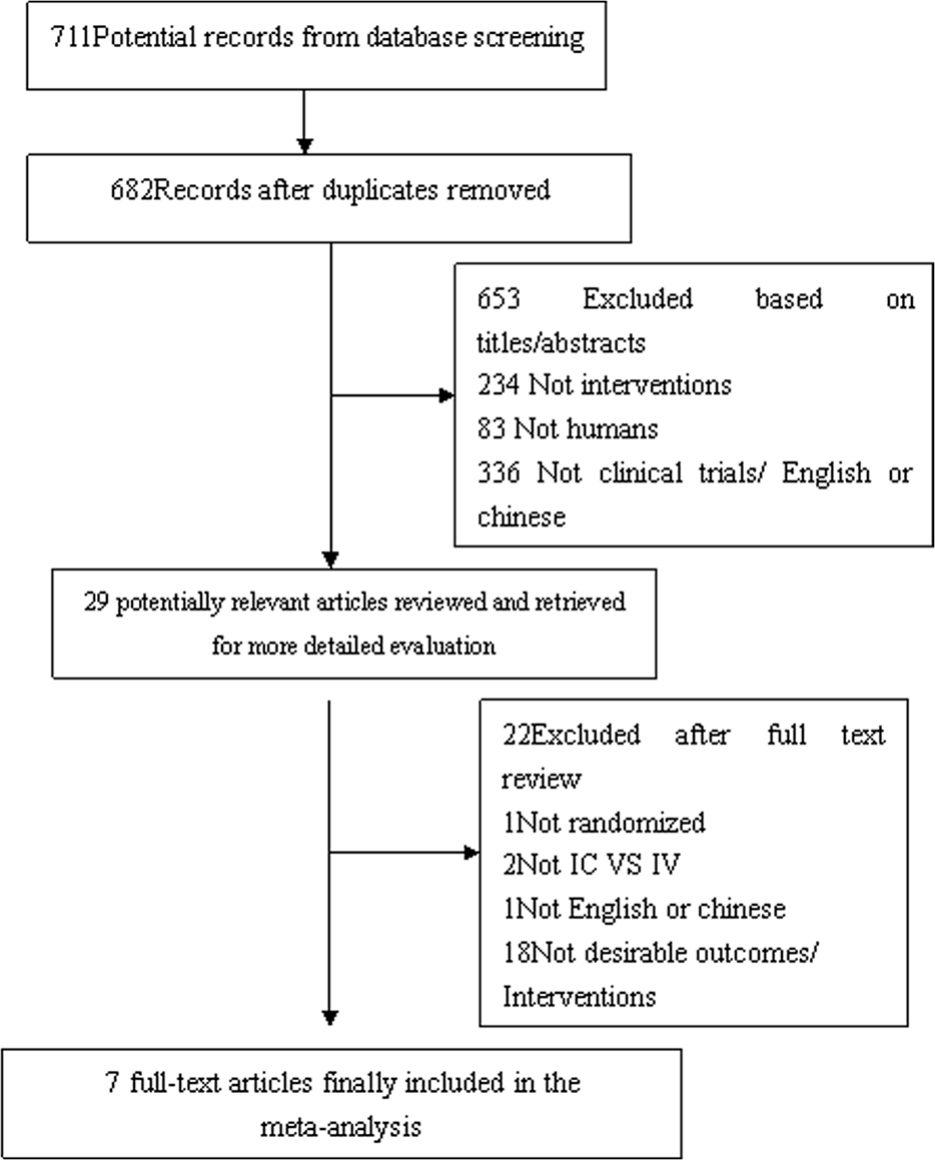
Study selection diagram.

We identified 711 potentially relevant articles from the electronic databases. After excluding duplicate research and screening the titles and abstracts of all potential articles, 29 potentially relevant articles were reviewed in full. After further evaluation, a total of 7 publications were included in the analysis. The flow diagram of the study process is shown in Fig 2.

The baseline characteristics of the 7 included RCTs are detailed in Tables 2 and 3. The studies involved 1,027 patients. All subjects who underwent PCI suffered from ACS. Approximately 76% of the enrolled patients were male; 96% had STEMI; approximately 26% of the patients had a diagnosis of diabetes mellitus; and more than 87% of the patients presented with TIMI grade 0–1 flow before PCI. The composition of the target vessel was not significantly different between the IC and IV treatment groups.

**Table 2:** Characteristics of patients and interventions in included studies.

Studies	Location	NO. of patients	Subjects	Ages(years)/male(%)	Intervention IC IV		Follow-up
Tian et al.2013	China	453	STEMI	64.6±11.9/81	IC bolus of tirofiban (10 ug.kg^−^) plus maintenance infusion (0.15ug.kg^−1^.min^−1^) for 24–36 h.	IC bolus of saline (10 ml) plus maintenance infusion of tirofiban (0.15ug.kg^−1^.min^−1^) for 24–36 h.	30 days 6 months
Candemir et.al,2012	Turkey	56	STEMI	69.4±8.6/59	IC high-dose bolus of tirofiban (25 ug.kg^−^) plus maintenance infusion (0.15ug. kg^−1^.min^−1^) for 24 h.	IV high-dose bolus of tirofiban (25 ug.kg^−^) plus maintenance infusion (0.15ug.kg^−1^.min^−1^) for 24 h.	In-hospital 30 days
Kırma et.al,2012	Turkey	49	STEMI	57.0±8.3/90	IC high-dose bolus of tirofiban (25 ug.kg^−1^) only	IV high-dose bolus of tirofiban (25 ug.kg^−^) plus maintenance infusion(18hr at 0.15ug.kg^−1^.min^−1^)	In-hospital 6 months
Erdim et.al,2010	Turkey	84	STEMI	55.0±12.0/90	IC bolus dose of tirofiban 10mcg.kg^−^ before angioplasty of infarctrelated artery, followed by a 36 hours of IV infusion at 0.15 mcg.kg^−1^.min^−1^	IV bolus dose of tirofiban 10 Mcg.kg^−^ before angioplasty of infarctrelated artery, followed by a 36 hours of IV infusion at 0.15 mcg.kg^−1^.min^−1^	In-hospital 6 months
Yan et al.2010	China	216	STEMI	58.1±14.2/73	Bolus administration of tirofiban through the aspiration catheter (500 μg) over a period of 3 minutes, then intravenous tirofiban (0.1 μg·kg-1·min-1) for 12 hours.	Noly intravenous tirofiban (0.1 μg·kg-1·min-1) for 12 hours.	In-hospital 9 months
Wu et.al,2008	China	115	ACS (63%STEMI)	75.0±2.0/55	IC bolus of tirofiban (10ug.kg^−^ over 3 minutes),then 36-hour IV infusion(0.15ug.kg^−1^.min^−1^)	IV bolus of tirofiban (10ug.kg^−1^ over 3 minutes), then 36-hour IV infusion(0.15ug.kg^−1^.min^−1^)	In-hospital 30 days
Yang et.al,2007	China	54	STEMI	58.8±12.6/79	IC bolus of tirofiban (10ug.kg^−^) before first balloon inflation, then 36-hour IV infusion(0.15ug.kg^−1^.min^−1^)	IV bolus of tirofiban (10ug.kg^−^) before angiography, then 36-hour IV infusion(0.15ug.kg^−1^.min^−1^)	In-hospital 30 days

STEMI = ST-elevation myocardial infarction; ACS = acute coronary syndrome; IC = intracoronary, IV = intravenous.

**Table 3:** Clinical and Procedural characteristics in the overall population.

Variables	IC	IV
Number	518	509
Male(%)	396(76.4)	385(75.6)
STEMI(%)	496(95.8)	488(95.9)
NSTEACS(%)	22(4.2)	21(4.1)
infarction localization (Anterior) (%)	73(14.1)	58(11.4)
Preinfarction angina pectoris(%)	32(6.2)	26(5.1)
Prior MI(%)	24(4.6)	25(4.9)
Hypertension(%)	219(42.3)	220(43.2)
Diabetes Mullites(%)	124(23.9)	140(27.5)
Cigarette smoking(%)	273(52.7)	261(51.2)
Medical therapy		
Aspirin(%)	510(98.5)	502(98.6)
Clopidogrel(%)	516(99.6)	508(99.8)
Target Vessel		
LM(%)	12(2.3)	10(2.0)
LAD(%)	245(47.3)	252(49.5)
RCA(%)	168(32.4)	161(31.6)
CX(%)	67(12.9)	64(12.6)
Thrombus in culprit vessel(%)	251(48.5)	252(49.5)
TIMI flow		
Before PCI (Grade 0–1), n (%)	457(88.2)	445(87.4)
After PCI (Grade 3), n (%)	474(91.5)	436(85.6)

STEMI = ST-elevation myocardial infarction; NSTEACS = Non ST-Elevation Acute Coronary Syndrome; MI = myocardial infarction; LM = left main; LAD = Left anterior descending; RCA = Right coronary artery; CX = Circumflex artery; TIMI = thrombolysis in myocardial infarction; PCI = percutaneous coronary intervention; IC = intracoronary, IV = intravenous.

All of the patients in these trials received tirofiban therapy during PCI, irrespective of the initial assignment. The initial bolus was delivered either via the IC or IV route, depending on the group. An injection of tirofiban was administered after the completion of coronary angiography, but immediately before angioplasty and/or stenting of an infarct-related artery in both groups. All patients received standard pharmacological therapy, including heparin, aspirin and clopidogrel. Seven studies [29–35] included a short-term follow-up (in-hospital and 30 days). The follow-up duration in four studies [32–35] was six to nine months (Table 2). All analysis indexes were detailed by S1 Table. No differences were observed in the baseline characteristics of the IC and IV administration groups.

### Efficacy Analysis Results

**Fig 3:**
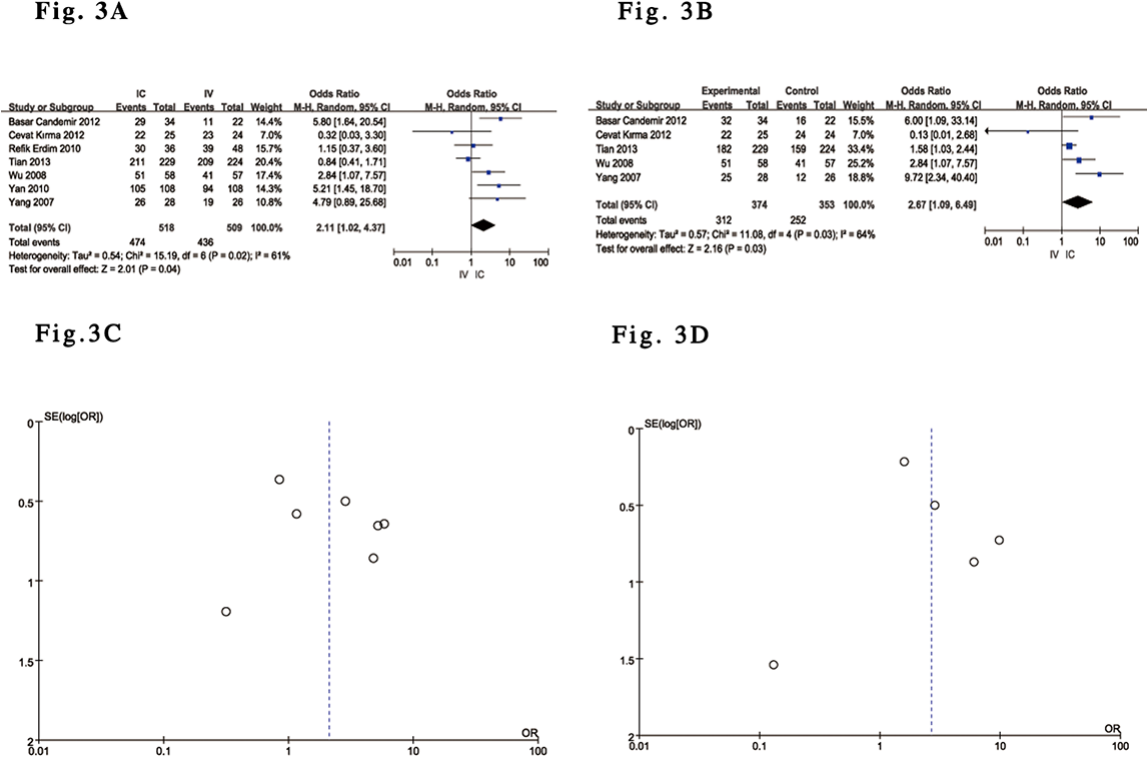
Forest plot of complete perfusion and TMP grade 3 in ACS patients. (A). Forest plot for increasing the frequency of complete perfusion based on a random-effects model in ACS patients treated with IC vs. IV administration of tirofiban. (B). Forest plot for increasing the frequency of TMP grade 3 based on a random-effects model in ACS patients treated with IC vs. IV administration of tirofiban. (C). Funnel plot of complete perfusion used to identify evidence of publication bias. (D). Funnel plot of TMP grade 3 used to identify evidence of publication bias.

**Fig 4:**
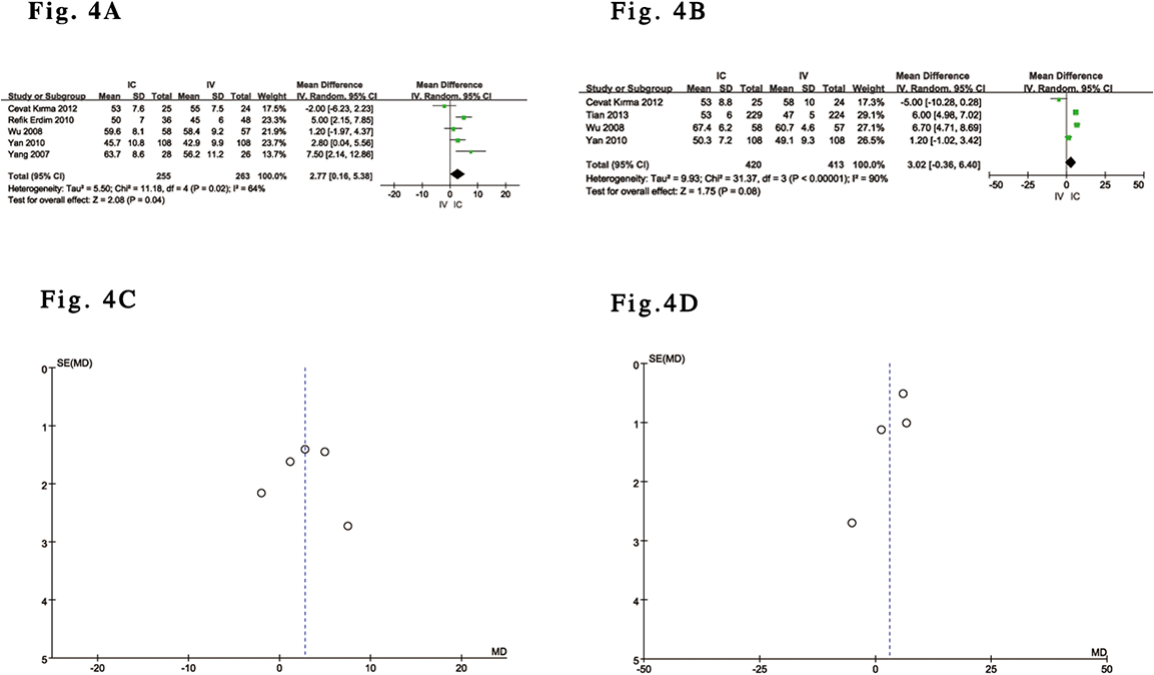
Forest plot of LVEF in ACS patients with IC vs. IV administration of tirofiban. (A). Forest plot for in-hospital LVEF based on a random-effects model in ACS patients with IC vs. IV administration of tirofiban. (B). Forest plot for LVEF over a medium-term follow-up, based on a random-effects model in ACS patients with IC vs. IV administration of tirofiban. (C). Funnel plot of in-hospital LVEF used to identify evidence of publication bias. (D). Funnel plot of LVEF over a medium-term follow-up used to identify evidence of publication bias.

**Fig 5:**
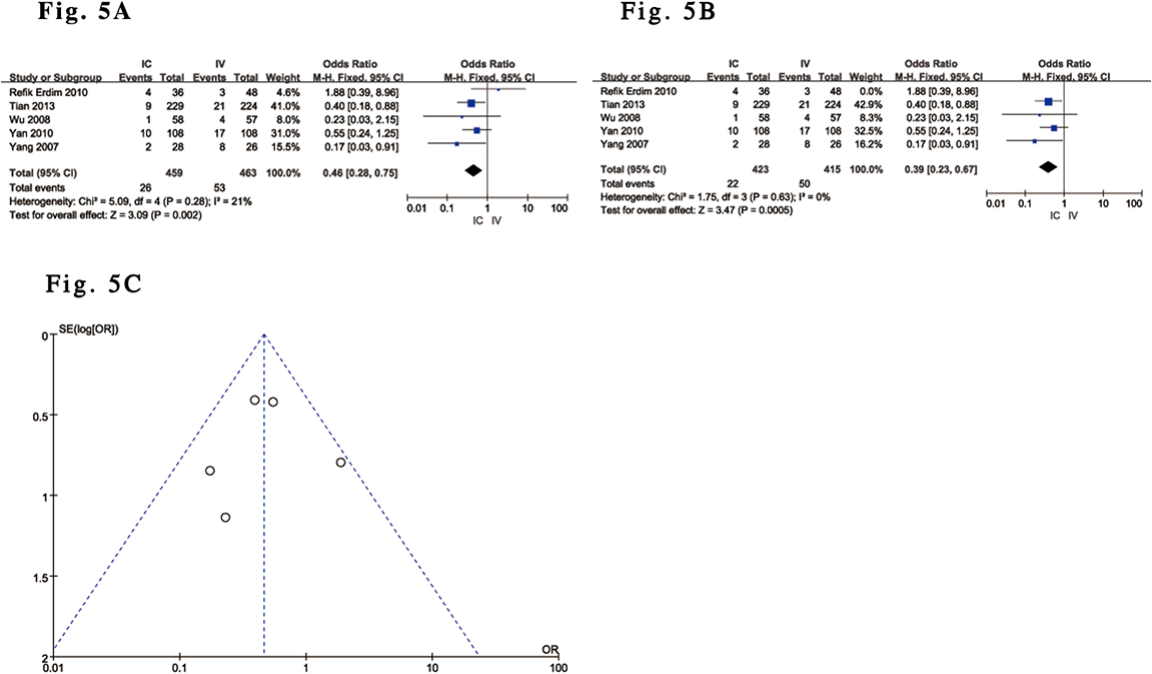
Forest plot of MACE in ACS patients with IC vs. IV administration of tirofiban. (A). Forest plot for total MACE based on a fixed-effects model in ACS patients with IC vs. IV administration of tirofiban. (B). Forest plot for MACE based on a fixed-effects model excluding one retrospective trial in ACS patients with IC vs. IV administration of tirofiban. (C). Funnel plot of MACE used to identify evidence of publication bias.

All of the included trials [29–35] reported the effects of IC versus IV administration of tirofiban after complete perfusion (TIMI grade 3 flow) following PCI. Five trials reported the effect of IC versus IV administration of tirofiban on TMP grade 3[29–31,33,35] and in-hospital LVEF [29,30,32–34]. The pooled results showed a significant difference in complete perfusion and TMP grade 3 after PCI as well as LVEF in in-hospital patients with ACS undergoing PCI who received IC tirofiban versus controls who received IV administration. Compared with IV tirofiban, IC tirofiban significantly increased the frequency of complete perfusion (OR 2.11; 95% CI 1.02 to 4.37; P = 0.04, I^2^ = 61%) and TMP grade 3 (OR 2.67; 95% CI 1.09 to 6.49; P = 0.03, I^2^ = 64%) after PCI based on a random-effects model (Fig 3). In other words, IC tirofiban was able to significantly decrease the frequency of the ‘no-reflow’ and ‘slow-flow’ phenomena after PCI. Funnel plot analysis of 7 included trials addressing complete perfusion and 5 included trials addressing TMP grade 3 did not suggest the presence of publication bias (Fig 3C and 3D). Although pooled analysis with a random-effects model also showed a significant difference in in-hospital LVEF between the two groups (MD 2.77; 95% CI 0.16 to 5.38; P = 0.04, I^2^ = 64%) (Fig 4A), the outcome of the analysis with a random-effects model [30,33–35] did not reveal any significant difference in LVEF over a relatively medium-term follow-up (30 days to 9 months) (MD 3.02; 95% CI -0.36 to 6.40; P = 0.08, I^2^ = 90%) (Fig 4B), and the associated Funnel plot analysis did not suggest the presence of publication bias (Fig 4C and 4D). Compared with IV administration, the overall outcomes from 5 of the included RCTs [29,30,32,34,35] based on a fixed-effects model suggested that IC tirofiban was associated with a relative reduction in MACE between the two groups of 54% (OR 0.46, 95% CI: 0.28 to 0.75; P = 0.002, I^2^ = 21%) (Fig 5A), and the outcome data were similar to the above outcome from 4 of the included trials [29,30,34,35] (OR 0.39, 95% CI: 0.23 to 0.67; P = 0.0005, I^2^ = 0%) (Fig 5B), with the exception of one retrospective study (32). Again, the outcomes regarding MACE according to a random-effects model were consistent with the above main analyses. Funnel plot analysis of 5 of the included trials addressing MACE did not suggest the presence of publication bias (Fig 5C).

**Fig 6:**
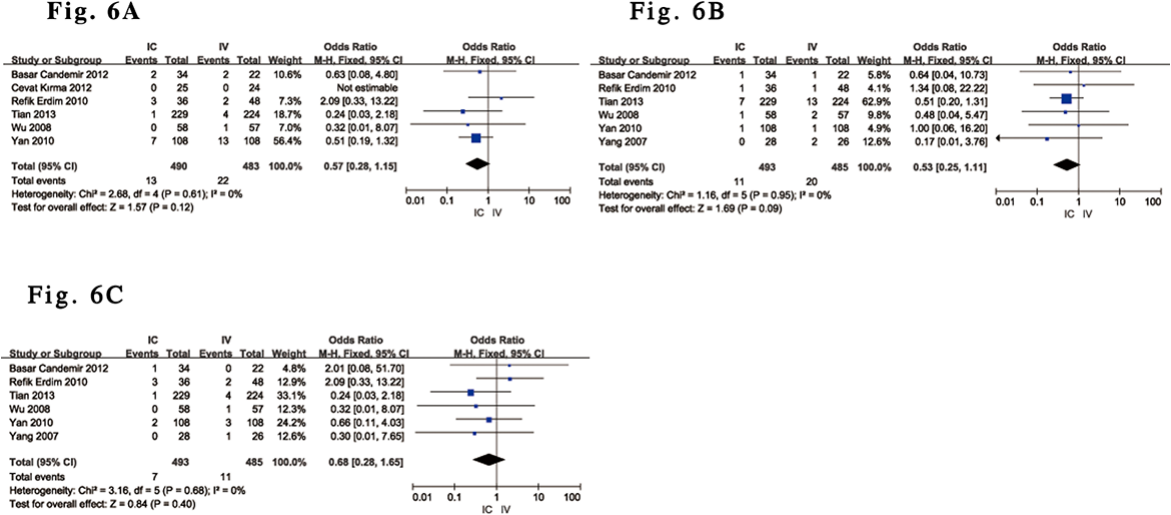
Forest plot of TVR, death and reinfarction in ACS patients treated with IC vs. IV administration of tirofiban. (A). Forest plot for TVR based on a fixed-effects model in ACS patients treated with IC vs. IV administration of tirofiban. (B). Forest plot for death based on a fixed-effects model in ACS patients treated with IC vs. IV administration of tirofiban. (C). Forest plot for reinfarction based on a fixed-effects model in ACS patients treated with IC vs. IV administration of tirofiban.

The pooled results indicated that TVR [30–35], death [29–32,34,35] and reinfarction [29–32,34,35] were not significantly reduced (P = 0.12, P = 0.09 and P = 0.40, respectively) in ACS subjects undergoing PCI who received IC tirofiban compared with controls who received IV administration (Fig 6). There was no heterogeneity detected among studies (all I^2^ = 0%), and the associated Funnel plot analysis did not suggest the presence of publication bias.

### Safety Analysis Results

**Fig 7:**
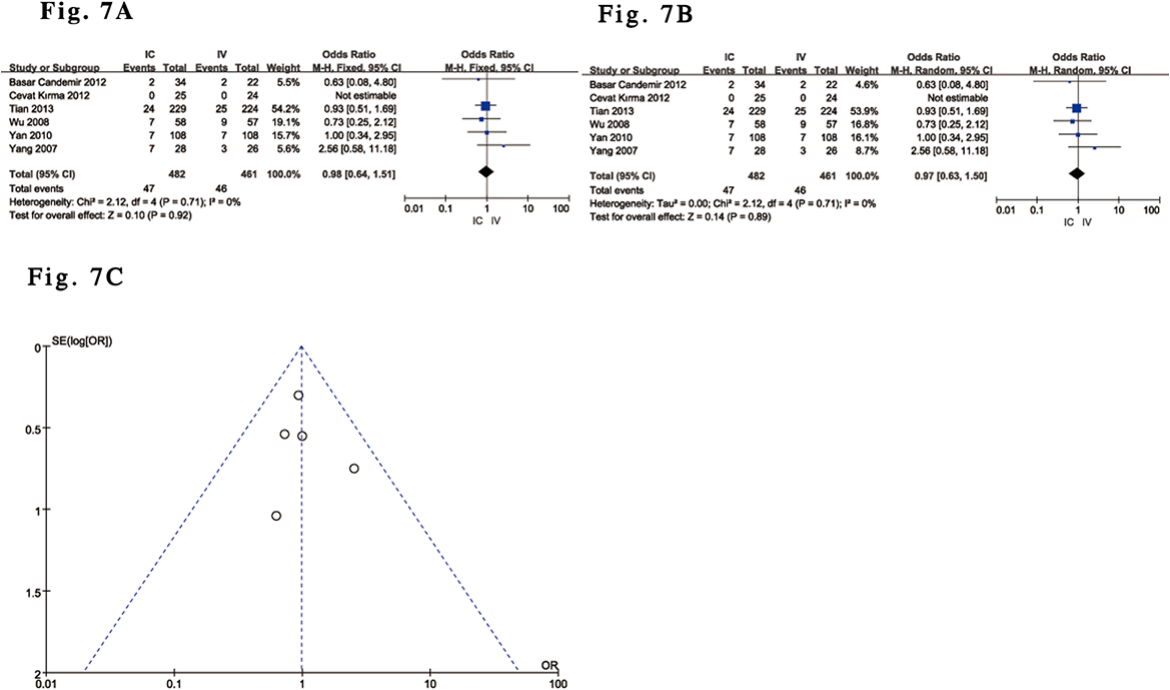
Forest plot of bleeding events in ACS patients treated with IC vs. IV administration of tirofiban. (A). Forest plot for bleeding events based on a fixed-effects model in ACS patients treated with IC vs. IV administration of tirofiban. (B). Forest plot for bleeding events based on a random-effects model in ACS patients treated with IC vs. IV administration of tirofiban. (C). Funnel plot of bleeding events used to identify evidence of publication bias.

Six trials [29–31,33–35] with 943 patients were included in which adverse drug reactions to tirofiban were reported, such as bleeding events, which were defined according to the TIMI criteria. All bleeding events were included, whether they were major or minor. The pooled results with a fixed-effects model showed no significant difference in the incidence of short-term bleeding events in ACS patients undergoing PCI who were treated with either IC or IV tirofiban (OR 0.98; 95% CI 0.64 to1.51; P = 0.92, I^2^ = 0%) (Fig 7A), and the outcomes with a random-effects model were consistent with the above results (OR 0.97; 95% CI 0.63 to1.50; P = 0.89) (Fig 7B). Funnel plot analysis of 6 of the included trials addressing bleeding events did not suggest the presence of publication bias (Fig 7C).

### Sensitivity analysis

**Fig 8:**
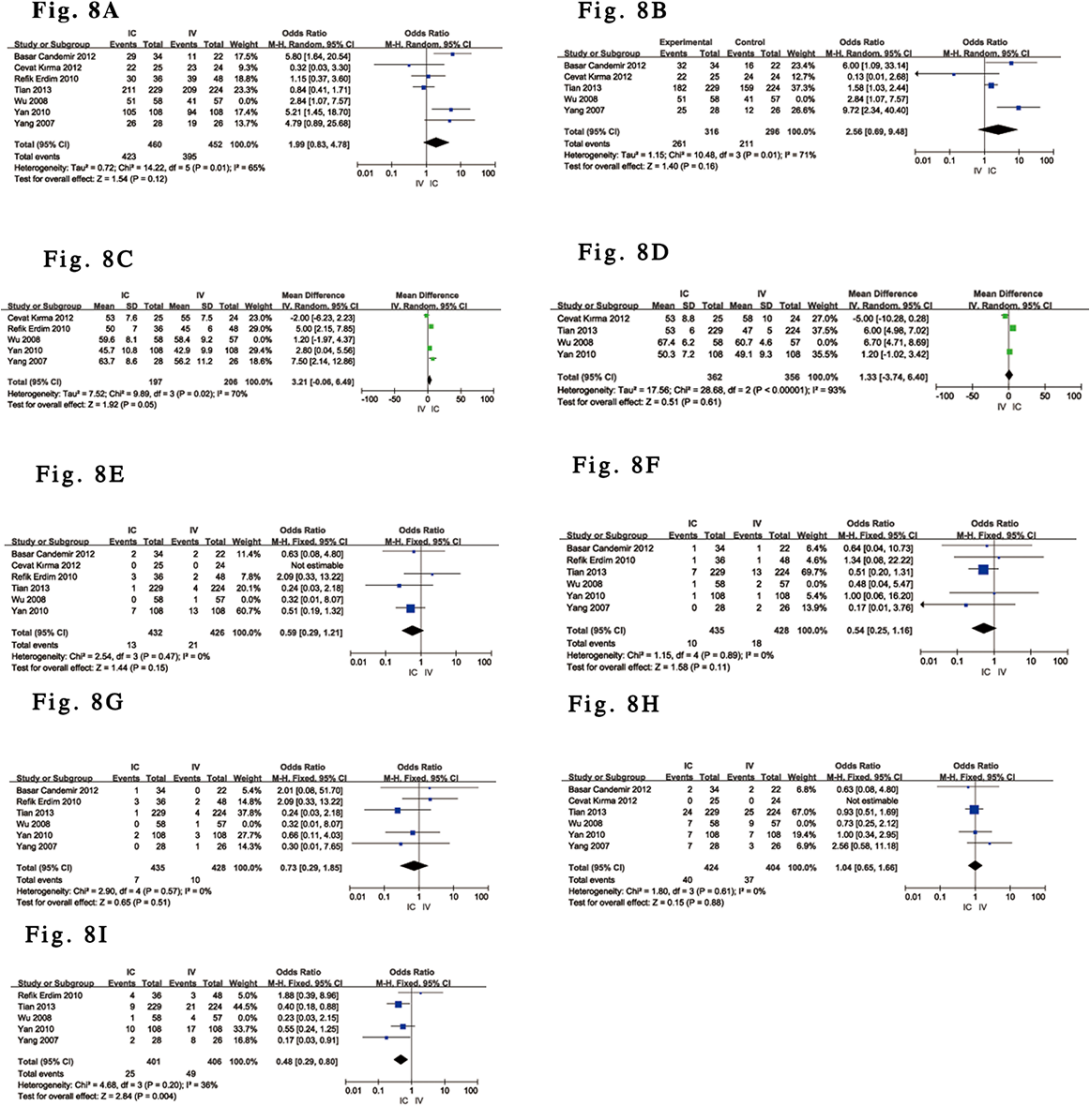
Forest plot for endpoints in STEMI patients treated with IC vs. IV administration of tirofiban. (A). Forest plot for TIMI grade 3 flow based on a random-effects model in STEMI patients treated with IC vs. IV administration of tirofiban. (B). Forest plot for TMP grade 3 based on a random-effects model in STEMI patients treated with IC vs. IV administration of tirofiban. (C). Forest plot for in-hospital LVEF based on a random-effects model in STEMI patients treated with IC vs. IV administration of tirofiban. (D). Forest plot for medium-term LVEF based on a random-effects model in STEMI patients treated with IC vs. IV administration of tirofiban. (E). Forest plot for TVR based on a fixed-effects model in STEMI patients treated with IC vs. IV administration of tirofiban. (F). Forest plot for death based on a fixed-effects model in STEMI patients treated with IC vs. IV administration of tirofiban. (G). Forest plot for reinfarction based on a fixed-effects model in STEMI patients treated with IC vs. IV administration of tirofiban. (H). Forest plot for bleeding events based on a fixed-effects model in STEMI patients treated with IC vs. IV administration of tirofiban. (I). Forest plot for MACE based on fixed-effects model in STEMI patients treated with IC vs. IV administration of tirofiban.

Sensitivity analyses of the RCTs [29,31–35] were conducted to evaluate the efficacy and safety of IC versus IV tirofiban in patients with ST-elevation myocardial infarction (STEMI) undergoing PCI. However, in the cases with STEMI and PCI, although no significant differences were detected between the effects of an IC or IV tirofiban bolus on complete perfusion after PCI [29,31–35], TMP grade 3 [29,31,33,35], in-hospital LVEF [29,32–34] and medium-term follow-up LVEF [33–35], TVR [31–35], death [29,31,32,34,35], reinfarction [29,31,32,34,35], and bleeding events [29,31,33–35] (P = 0.12, P = 0.16, P = 0.05 and P = 0.61, P = 0.15, P = 0.11, P = 0.51, and P = 0.88, respectively), the pooled data [29,32,34,35] with a fixed-effects model showed that MACE was significantly reduced by IC tirofiban in STEMI patients compared with IV administration (OR 0.48; 95% CI 0.29 to 0.80; P = 0.004, I^2^ = 36%, heterogeneity P = 0.20). All of the analysis outcomes are detailed in Fig 8. The outcomes regarding MACE based on a random-effects model were also consistent with the above main analyses, and none of the Funnel plot analyses addressing the above outcomes suggested the presence of publication bias.

## Discussion

Despite the substantial progress that has been made in recent decades regarding the treatment of ACS, including thrombus aspiration and routine stenting, questions have been raised concerning the potential benefit of GPIs in ACS patients undergoing PCI. Positive benefits of tirofiban were observed in ACS patients undergoing PCI who received IC tirofiban compared with controls who received IV administration. These benefits included an increase in the incidence of complete perfusion and TMP grade 3 after PCI and a reduction in MACE, although there was no significant benefit in terms of medium-term follow-up LVEF, TVR, death and reinfarction. Regarding safety end points, there were no differences between the two groups in the incidence of bleeding events.

The rationale for IC administration of tirofiban during PCI is to achieve a higher drug concentration in the area of the culprit lesion and in the distal bed of the culprit vessel. Compared with IV delivery of tirofiban, a higher drug concentration should result from IC delivery, leading to a greater procedural success rate (e.g., TIMI grade 3 flow) [36,37]. The most important effect is that a high local concentration of GPI has a thrombolytic effect, which improves TIMI flow [23,38,39].Therefore, it is logical to conclude that IC tirofiban yields better receptor occupancy and additional thrombolytic effects compared with IV administration. Consistent with this pharmacologic mechanism, the present meta-analysis found that TIMI flow and TMP flow were significantly increased after the initial IC tirofiban bolus compared with IV administration during PCI in patients with ACS.

Impaired TIMI flow or myocardial reperfusion is closely related to increased MACE in ACS patients undergoing PCI [40,41]. By substantially increasing TIMI flow and TMP grade 3 in ACS patients, IC administration of tirofiban can reduce MACE. A previous analysis of 1,346 patients found that treatment with IC GPIs was associated with significant benefits in terms of MACE compared with IV administration (OR 0.48; 95% CI 0.31 to 0.76; P<0.005) [24]. Consistent with the above outcomes, this meta-analysis clearly demonstrated that IC administration of tirofiban leads to a reduction in MACE for ACS patients, either including or excluding the one retrospective trial [32].

The findings of this meta-analysis are consistent with a previous meta-analysis evaluating the clinical benefits of IC GPIs compared with IV administration. The magnitude of the effects observed in this study are also comparable [25]. The analysis showed that the effects of tirofiban on medium-term follow-up LVEF, TVR, death and reinfarction were not significantly different between the two groups, regardless of whether the one retrospective trial [32] was included.

All GPIs may increase the risk of bleeding because of their antiplatelet activity and antithrombotic properties. Therefore, in the present meta-analysis, the safety end point of the IC bolus was defined as the occurrence of bleeding events, which were not significantly different between the two groups. This is not surprising because more caution is currently applied to the dosing of antiplatelet and antithrombotic agents, and closer attention is paid to the management of patients. In patients with similar baseline characteristics who were randomized to an IC or IV group, the incidence of bleeding events was low, with no significant difference being noted between the two groups.

However, inconsistent with the recently reported effects of GPIs in STEMI patients undergoing PCI [23,24], this meta-analysis showed that there was no significant difference in LVEF, TIMI grade 3 flow, TMP grade 3 and TVR, with the exception of MACE, between IC and IV tirofiban administration. This was most likely due to the small sample size of the RCTs included in this meta-analysis and the fact that different pharmacologic regimens were used in the studies.

Although this meta-analysis revealed positive outcomes associated with IC tirofiban, including an improvement of TIMI flow, TMP flow and MACE for ACS patients, there was no change in the following outcome measures: medium-term follow-up LVEF, TVR, death and reinfarction. Nevertheless, the noted improvements still represent substantial benefits for the recovery of ACS patients, especially considering the simplicity of the change in the drug administration protocol. Such benefits are also achieved solely by changing the initial bolus, without altering the maintenance infusion. Furthermore, because there are no time restrictions surrounding the catheterization procedure, the cost to patients is similar for both the IC and IV bolus administration of tirofiban. Taken together, these benefits suggest that even though the observed improvements are limited to TIMI flow and MACE, the use of IC over IV tirofiban can still be justified. Large-scale, high-quality RCTs designed to evaluate cost-effectiveness are clearly required to further investigate the merits of IC versus IV bolus administrations of tirofiban.

Compared with previous meta-analyses, this meta-analysis offers several strengths. First, to our knowledge, this is the first meta-analysis to directly compare the effects of IC and IV administration of the initial tirofiban bolus in ACS patients undergoing PCI. Second, we used a predesigned protocol for the literature search, study selection and data synthesis. All methods closely adhered to the PRISMA guidelines [28]. We assessed heterogeneity using the Q test and I^2^ and pooled data using a random-effects model if substantial heterogeneity was observed (I^2^≥ 50% and heterogeneity P≤0.1). We also conducted sensitivity analyses to test the sensitivity of the results to the systematic review and meta-analysis methodology. Finally, the risk of bias was assessed using the recommended Cochrane collaboration’s tool. The previous meta-analyses [24,25,42] either failed to assess the quality of studies or only used the Jadad scale, which does not consider allocation concealment and is not recommended by the Cochrane Handbook for Systematic Reviews of Interventions [43]. Therefore, the results of the methodological quality assessment performed in our study are more credible.

The limitations of this study deserve comment. First, because this meta-analysis only included published data in English or Chinese, some potential for bias is present. Second, all of the included RCTs lacked long-term data (≥12 months), and in some cases, certain outcomes could not be assessed, even at the 6-month follow-up. Finally, different follow-up times and therapy doses could also influence conclusions about the differences between the IC and IV groups.

## Conclusions

This meta-analysis supports the use of IC over IV administration of tirofiban for ACS to improve TIMI flow, TMP flow and MACE. However, there was no significant difference in the risk of bleeding complications between the two groups.

## Supporting Information

S1 Table Clinical events in follow-up. (DOC)
