# Supplementary material for: Efficacy and Safety of Intracoronary versus Intravenous Administration of Tirofiban during Percutaneous Coronary Intervention for Acute Coronary Syndrome: A Meta-Analysis of Randomized Controlled Trials
Source: PLoS One. 2015 Jun 11;10(6):e0129718. doi: 10.1371/journal.pone.0129718 (PMC4465926; doi:10.1371/journal.pone.0129718)
Supplement: S1 Table — (DOC) [file pone.0129718.s001.doc]

S1 Table. Clinical events in follow-up

| Events | Tian et al.2013 | | Candemir et.al,2012 | | Kırma et.al,2012 | | Erdim et.al,2010 | | Yan et al.2010 | | Wu et.al,2008 | | Yang et.al,2007 | |
| --- | --- | --- | --- | --- | --- | --- | --- | --- | --- | --- | --- | --- | --- | --- |
|  | IC  (n=229) | IV  (n=224) | IC  (n=34) | IV  (n=22) | IC  (n=25) | IV  (n=24) | IC  (n=36) | IV  (n=48) | IC  (n=108) | IV  (n108) | IC  (n=58） | IV  (n=57) | IC  (n=28) | IV  (n=26) |
| TIMI flow After PCI (Grade 3), n | 211 | 209 | 29 | 11 | 22 | 23 | 30 | 39 | 105 | 94 | 51 | 41 | 26 | 19 |
| TMP grade 3 | 182 | 159 | 32 | 16 | 22 | 24 | NA | NA | NA | NA | 51 | 41 | 25 | 12 |
| LVEF(%,Ｘ±Ｓ) |  |  |  |  |  |  |  |  |  |  |  |  |  |  |
| In-hospital | NA | NA | NA | NA | 53±7.6 | 55±7.5 | 50±7 | 45±6 | 45.7±10.8 | 42.9±9.9 | 59.6±8.1 | 58.4±9.2 | 63.7±8.6 | 56.2±11.2 |
| In follow-up(30days or 6 months) | 53±6 | 47±5 | NA | NA | 53±8.8 | 58±10 | NA | NA | 50.3±7.2 | 49.1±9.3 | 67.4±6.2 | 60.7±4.6 | NA | NA |
| MACE | 9 | 21 | NA | NA | NA | NA | 4 | 3 | 10 | 17 | 1 | 4 | 2 | 8 |
| TVR | 1 | 4 | 2 | 2 | 0 | 0 | 3 | 2 | 7 | 13 | 0 | 1 | NA | NA |
| Death | 7 | 13 | 1 | 1 | NA | NA | 1 | 1 | 1 | 1 | 1 | 2 | 0 | 2 |
| Reinfarction | 1 | 4 | 1 | 0 | NA | NA | 3 | 2 | 2 | 3 | 0 | 1 | 0 | 1 |
| Bleeding | 24 | 25 | 2 | 2 | 0 | 0 | NA | NA | 7 | 7 | 7 | 9 | 7 | 3 |

TIMI= thrombolysis in myocardial infarction;PCI= percutaneous coronary intervention; TMP=TIMI myocardial perfusion; LVEF= left ventricular ejection fraction; MACE= major adverse cardiovascular events; TVR= target vessel revascularization; IC = intracoronary, IV=intravenous; NA= not available
